# Adult Pelvic Sarcomas: A Heterogeneous Collection of Sarcomas?

**DOI:** 10.1080/13577140410001679211

**Published:** 2004-03

**Authors:** Claudia M. G. Keyzer-Dekker, Richard G. Houtkamp, Johannes L. Peterse, Frits Van Coevorden

**Affiliations:** 1 Department of Surgery Netherlands Cancer Institute Plesmanlaan 121 Amsterdam 1066 CX The Netherlands; 2 Department of Pathology Netherlands Cancer Institute Amsterdam The Netherlands

## Abstract

*Introduction*. Adult pelvic soft tissue sarcomas are a rare group of heterogeneous malignancies. These sarcomas differ from extremity and trunk soft tissue sarcomas in presentation, characteristics and response to treatment.

*Methods*. A retrospective analysis of patient and tumor characteristics, treatment and prognosis and prognostic factors was performed.

*Results*. Between 1977 and 1997, a total of 33 adult patients with soft tissue sarcomas involving the pelvis but excluding uterine leiomyosarcoma were identified. Leiomyosarcomas (18), including six GIST, and rhabdomyosarcomas (eight) were the most commonly seen tumors. At first presentation, nine patients already had metastases. The mean follow-up was 52 months (1–200). Recurrences developed in 15 of the 24 cases (63%) with tumors without metastases at first presentation; in six (25%) recurrence was locally only, in nine distant metastases occurred. The nine patients with metastatic disease at first presentation died of the disease, while eight of the 24 patients with localized disease at presentation died. One patient died of an unrelated cause, four were alive with disease, and 11 patients were alive and free of disease. The only identifiable prognostic factor of disease-free interval and overall survival was histological grade.

*Conclusion*. Soft tissue sarcomas of the pelvis appear to be associated with increased rate of metastasis at the time of diagnosis and higher rates of local recurrence. In this study, multi-modality treatment for most primary tumors did not show a significant benefit in recurrence rate, DFI and OST, when compared to single modality approach. Although the number of patients in this study is small, and different types of sarcomas were studied, the only identifiable predictor for survival was low histological grade of the tumors. The differences of this heterogeneous group of pelvic sarcomas with retroperitoneal, trunk and extremity sarcomas should be taken into consideration in the management of these sarcomas.


*Sarcoma*, March 2004, *VOL. 8, NO. 1, 19–24*

Taylor & Francis healthsciences

ORIGINAL ARTICLE

### Adult pelvic sarcomas: a heterogeneous collection of sarcomas?

CLAUDIA M.G. KEYZER-DEKKER[1], RICHARD G. HOUTKAMP[1], JOHANNES L. PETERSE[2] & FRITS VAN COEVORDEN[1]

*Departments of [1]Surgery and [2]Pathology, Netherlands Cancer Institute, Amsterdam, The Netherlands*

**Abstract**

*Introduction*. Adult pelvic soft tissue sarcomas are a rare group of heterogeneous malignancies. These sarcomas differ from extremity and trunk soft tissue sarcomas in presentation, characteristics and response to treatment.

*Methods*. A retrospective analysis of patient and tumor characteristics, treatment and prognosis and prognostic factors was performed.

*Results*. Between 1977 and 1997, a total of 33 adult patients with soft tissue sarcomas involving the pelvis but excluding uterine leiomyosarcoma were identified. Leiomyosarcomas (18), including six GIST, and rhabdomyosarcomas (eight) were the most commonly seen tumors. At first presentation, nine patients already had metastases. The mean follow-up was 52 months (1–200). Recurrences developed in 15 of the 24 cases (63%) with tumors without metastases at first presentation; in six (25%) recurrence was locally only, in nine distant metastases occurred. The nine patients with metastatic disease at first presentation died of the disease, while eight of the 24 patients with localized disease at presentation died. One patient died of an unrelated cause, four were alive with disease, and 11 patients were alive and free of disease. The only identifiable prognostic factor of disease-free interval and overall survival was histological grade.

*Conclusion*. Soft tissue sarcomas of the pelvis appear to be associated with increased rate of metastasis at the time of diagnosis and higher rates of local recurrence. In this study, multi-modality treatment for most primary tumors did not show a significant benefit in recurrence rate, DFI and OST, when compared to single modality approach. Although the number of patients in this study is small, and different types of sarcomas were studied, the only identifiable predictor for survival was low histological grade of the tumors. The differences of this heterogeneous group of pelvic sarcomas with retroperitoneal, trunk and extremity sarcomas should be taken into consideration in the management of these sarcomas.

**Key words:** *sarcoma, pelvis, treatment*

### Introduction

Less than 5% of all sarcomas are located in the pelvic region and the majority of these are related to the genitourinary tract.[1,2] These sarcomas may not produce symptoms until they are large and have extensively invaded local tissues.[3] Local and distant recurrent disease is a major problem, resulting in a high disease-related mortality rate.[1] Previous studies have demonstrated that surgery offers the most effective treatment for adult sarcoma of the pelvis.[2,3] However, anatomical constraints of the pelvis and early involvement of adjacent structures often mean that excision margins in most cases are marginal.[3] Adjuvant radiotherapy and chemotherapy in adult patients with STS of the pelvis have not improved overall survival, but may have effect on local control.[2,3] This study of patients with STS of the pelvis was performed to evaluate the characteristics and the results of treatment.

### Methods

We collected data from all adult patients with a diagnosis of soft tissue sarcoma of the pelvic region from 1977 to 1997 treated at the NKI/AvL retrospectively.

These included sarcomas of the genitourinary and digestive tract as far as they are located around the pelvis or pelvic floor. Ovarian sarcomas and uterine sarcomas, including mixed Müllerian tumors, were excluded.

A total of 33 patients with soft tissue sarcomas in the pelvis were identified in the prospective cancer database in our institute. This represents 6% of the 1415 patients with soft tissue sarcomas seen at our center over the period of study. In 26 of the 33 patients, we were involved in the primary treatment.

The pathology was reviewed to obtain a uniform histological classification and grade, according to actual criteria.[4] Follow-up was noted from the last

Correspondence to: F. van Coevorden, MD PhD, Netherlands Cancer Institute, Plesmanlaan 121, 1066 CX Amsterdam, The Netherlands. Fax: +31-20-512-2554; E-mail: f.v.coevorden@nki.nl

1357-714X print/1369–1643 © 2004 Taylor & Francis Ltd
DOI: 10.1080/13577140410001679211

recorded visit in the patient's files. Survival time and disease-free interval were calculated from the date of the start of treatment.

### Results

### *Patient characteristics*

There were 12 females and 21 males, age ranging from 16 to 79 years (median age 49 years). The median age of females was 49 and for men 36 years (Table 1).

### *Tumor characteristics*

Histological proof of sarcoma was made by large core needle biopsy or incisional biopsy in 14 patients and by excisional biopsy in 19 patients.

The most common histological types were leiomyosarcoma (LMS) (18 patients, including six gastrointestinal stromal cell tumors of the rectum) and rhabdomyosarcoma (RMS) (eight, including four embryonal and four other sub-types). Two liposarcomas (LPS), two malignant fibrous histiocytomas (MFH), one dermatofibrosarcoma protuberans (DFSP), one epithelioid sarcoma (ECS) and one sarcoma not otherwise specified (SNOS) were seen.

The site of the tumor, relation to deep fascia, histological grade and presence or absence of metastases at presentation are shown in Table 2.

The median size of tumor was 5 cm (range 2–13 cm). Most tumors were related to the male (16) or female (eight) urogenital tract; eight tumors originated in or were adjacent to the rectal wall and one was in the pelvic venous plexus. Most tumors were deep to the fascia (30/33), were intermediate to high grade (31/33) and in nine patients metastatic disease was established at the moment of diagnosis (Table 2).

### *Treatment characteristics*

Primary therapy consisted of single modality treatment in 15 cases and multi-modality therapy in 18 patients, as specified in Table 3. Our institution was involved in (part of) the primary treatment in 26 of 33 patients.

Six of eight patients with RMS (including three with primary metastatic disease) were treated by multi-modality approach, as were nine patients with LMS (no primary metastatic disease). Two of the primary metastatic RMS patients were treated by single modality only (chemotherapy), as were the other nine patients with LMS (two with primary metastatic disease).

Primary metastatic disease was thus treated by multi-modality approach in four and by single modality approach in five patients. In these nine patients presenting with metastatic disease, surgery (four), radiotherapy (four) and chemotherapy (five) were applied almost equally.

All 24 patients with localized disease had surgical excision, combined with radiotherapy in 12 and chemotherapy in three patients.

Resection margins were wide in eight patients, marginal in 10 cases and intralesional (debulking) in 10 cases.

**Table 2.** *Localisation, relation to the fascia, grade and primary presentation*

| | |
|---|---|
| *Localisation* | |
| Bladder | 1 |
| (Para)rectal | 8 |
| (Para)testicular | 4 |
| Pelvic vessels | 1 |
| Prostate | 3 |
| Spermatic cord | 8 |
| Vulva | 8 |
| *Relation to fascia* | |
| Superficial | 3 |
| Deep | 30 |
| *Grade* | |
| Low | 2 |
| Intermediate | 14 |
| High | 17 |
| *Primary presentation* | |
| Local disease only | 24 |
| Primary metastasized | 9 |

**Table 1.** *Patients, median age, histological classification*

| | Total | Male | Female | Median age (years) |
|---|---|---|---|---|
| Patients (*N*) | 33 | 21 | 12 | |
| Median age (range) | | 36 (16–79) | 49 (36–76) | 49 (16–79) |
| Histology | | | | |
| Embryonal rhabdomyosarcoma | 4 | 4 | 0 | 17 |
| Other rhabdomyosarcoma | 4 | 4 | 0 | 20 |
| Leiomyosarcoma (non-GIST) | 12 | 7 | 5 | 59 |
| Leiomyosarcoma (GIST) | 6 | 4 | 2 | 51 |
| Malignant fibrous histiocytoma | 2 | 0 | 2 | 59 |
| Myxoid liposarcoma | 2 | 1 | 1 | 34 |
| Epithelioid sarcoma | 1 | 0 | 1 | 37 |
| Dermatofibrosarcoma | 1 | 0 | 1 | 49 |
| Sarcoma n.o.s. | 1 | 1 | 0 | 50 |

**Table 3.** *Histology, type of treatment, complications and survival*

| | Single disciplinary treatment | | Multi-disciplinary treatment | |
|---|---|---|---|---|
| Histology | RMS | 2 | RMS | 6 |
| | LMS | 9 | LMS | 9 |
| | Other | 4 | Other | 3 |
| Treatment | Surgery | 12 | Surgery/Radiotherapy | 11 |
| | Radiotherapy | 1 | Surgery/Chemotherapy | 4 |
| | Chemotherapy | 2 | Chemotherapy/Radiotherapy | 2 |
| | | | Surgery/radiotherapy/chemotherapy | 1 |
| Complications | Osteomyelitis (surg related) | 1 | Haematoma (surg related) | 3 |
| | Sepsis (chemo related) | 1 | Nausea (chemo related) | 1 |
| | | | Impotence (surg plus radio related) | 1 |
| Survival | NED | 5 *(33%)* | NED | 6 *(33%)* |
| | ED | 2 *(13%)* | ED | 2 *(11%)* |
| | DOD | 8 *(54%)* | DOD | 9 *(50%)* |
| | | | UND | 1 *(6%)* |

*Abbreviations:* RMS, rhabdomyosarcoma; LMS, leiomyosarcoma; NED, no evidence of disease; ED, evidence of disease; DOD, died of disease; UND, unrelated death.

Radiotherapy doses varied between 16 and 66 Gy, depending on the purpose of the treatment. As adjuvant therapy after resection with tumor-free margins, 60 Gy was considered sufficient. After incomplete surgery, radiotherapy with a boost up to 66 Gy was administered.

Chemotherapy treatment included administration of several drugs: actinomycin A, adriamycin, etoposide, ifosfamide and vincristine in different combinations. Primary chemotherapy was chosen as treatment modality in five of nine cases presenting with disseminated disease.

*Complications of treatment*

Primary treatment of these 33 patients was uneventful in 26 of 33 patients (79%). Single modality treatment (15 patients) was correlated to two eventful courses; one patient developed a sepsis under chemotherapy and in another patient osteomyelitis of the pubic bone occurred after radical vulvectomy. Multi-modality treatment (18 patients) was uneventful in 13 patients (72%). Nausea, impotence and three cases of hematoma were seen (Table 3).

*Local and distant failure*

Nine patients had metastatic disease at presentation. All progressed on treatment or responded for short periods. Of the 24 patients with localized disease at presentation, 15 (63%) recurred after primary treatment.

The two low-grade tumors (one LPS and one DFSP) did not recur or metastasize; 10 of 12 grade II (83%) and five of 10 grade III (50%) recurred.

Local recurrence as first presentation of recurrent disease was seen in six of the 24 patients. Multiple local recurrences were seen in one patient with a myxoid liposarcoma. After wide or marginal surgery, sarcoma recurred in 10 of 16 patients, and after intralesional surgery in five of eight patients. The average time to local recurrence was 19 months.

Pulmonary metastatic disease occurred in five of 24 patients, while in six patients extrapulmonary sites of metastases were seen as first manifestation of metastatic disease. The average time to pulmonary metastases was 13 months, and to extrapulmonary distant disease this was 22 months. Two patients with pulmonary metastases had resection of the metastases and one of these patients survived for more than 2 years.

Of the six patients with extrapulmonary metastases, two with GIST underwent partial liver resection and both survived for more than 5 years after liver resection.

One patient with extrapulmonary metastatic disease had a long-term survival (> 5 years) after combination of chemo- and radiotherapy. One of our patients who had multiple resections of metastatic disease is still alive without disease activity as a result of Imatinib treatment.

*Patients without metastases at presentation*

There was a difference in DFI between RMS (two patients, mean interval 9 months) and LMS (10 patients, mean interval 27 months) ($P = 0.36$). Table 4 shows the DFI in relation to histology, grade, surgical margins and treatment modality.

*Survival time and prognostic factors*

In this study, the actuarial 5-year survival was 33%. The median overall survival in this study was 45 months, range 1–200 months. At the last follow-up, 17 of 33 patients had died of the disease (DOD), four of 33 were alive with evidence of disease (ED), 11 alive without evidence of the disease (NED) and one patient died of unrelated cause (UND).

**Table 4.** *Disease-free interval and overall survival time (mean in months) in primary non-metastatic disease (24 patients) in relation to histology, grade, surgical margins and treatment modality*

| | Number of patients (recurrence/all) | DFI (months; average (range)) | OST (months; average (range)) |
|---|---|---|---|
| *Histology* | | | |
| RMS | 2/3 | 9 (3–16) | 99 (12–142) |
| LMS | 10/16 | 27 (3–140) | 94 (5–159) |
| Other | 3/5 | 20 (13–32) | 164 (18–200) |
| *Grade* | | | |
| Low | 0/2 | | 113 (112–115) |
| Intermediate | 10/12 | 31 (6–140) | 123 (12–200) |
| High | 5/10 | 8 (3–16) | 86 (5–142) |
| *Surgical margins* | | | |
| Intralesional | 5/8 | 14 (3–34) | 61 (5–112) |
| Marginal/wide | 10/16 | 28 (3–140) | 139 (12–200) |
| *Treatment modality* | | | |
| Single | 8/10 | 35 (6–140) | 149 (12–200) |
| Multi | 7/14 | 10 (3–20) | 81 (5–142) |

*Abbreviations:* DFI, disease-free interval; OST, overall survival time; RMS, rhabdomyosarcoma; LMS, leiomyosarcoma.

The mean overall survival time in 13 patients treated with surgery and radiotherapy was 62 months (range 5–112); for 15 patients treated with surgery only, the overall survival time was 116 months (range 9–200) ($P=0.73$). The mean disease-free interval after surgery and radiotherapy was 11 months, and this was 25 months ($P=0.39$) after surgery alone.

After surgery and radiotherapy, six of 13 patients had a recurrence; after surgery, 12 of 15 patients had a recurrence ($P=0.06$). The overall survival time in patients with localized disease was 105 months in patients (nine) without recurrence and 113 months with recurrence (15).

The influence of non-metastatic disease on survival was analysed for histology, grade, surgical margins and treatment modality (Table 4). None of the factors analysed was statistically significant.

### Discussion

Sarcomas located in the pelvic region are rare, the number of relevant studies are limited and the inclusion criteria show major differences, therefore comparisons with other studies are difficult.

In this study, LMS and RMS formed the majority of all cases. An analysis of two so different types of tumor has great limitations. RMS and LMS are seen at different decades of life. RMS is always a high grade tumor that presents in adolescents, often already with metastatic disease. Moreover, their treatment may differ since many of these young adults with RMS will be subjected to treatment according to childhood and young adolescent regimens. LMS, however, is often seen in adults, and presents usually as local disease only, on which the treatment often is focused.

Our study, however, compares well with a study of 43 patients treated in MSKCC.[2] The most common histological types in the MSKCC study were also LMS (17) and RMS (13), there were five low grade tumors and 31 high grade tumors (if we exclude the seven patients with kidney tumors in that study). In the MSKCC study nine of 43 presented with metastatic disease, almost all (8/9) with RMS. Where in that study only two of the nine patients survived (both RMS), all patients with primary metastatic disease in our study died with a mean OST of 17 months. Presentation with metastatic disease can therefore be concluded to be a poor prognostic factor.

Analogous to childhood RMS, treatment of RMS usually consists of a combination of chemotherapy and local treatment. Combination treatment in metastatic disease in our young adults, however, showed no ultimate beneficial effect. Chemotherapy-based treatment in the three others with local RMS only resulted in one long-term survivor, and death from disease 12 months after start of treatment in a second patient. Another long-term survivor with RMS had local combination treatment only. The value of chemotherapy-based treatment in this study thus remains unclear.

A possible major difference in treatment outcome, even in metastatic disease, however, is now emerging in another subgroup. As one of our patients has experienced, the introduction of tyrosine kinase inhibitors such as Imatinib may change the prognosis of patients with GIST even, or in particular, when this disease has metastasized.[18]

We have analyzed the effect of single versus multi-modality treatment on recurrence rate, disease-free interval and overall survival time. The patients in these two treatment modality groups were comparable except for tumor grade; there were more grade 2 tumors in the single modality treatment group (10/15) and more grade 3 tumors in the multi-modality treatment group (13/18). But tumor grade as a single prognostic factor did not reveal statistical significant differences in survival.

In non-metastatic disease, all patients were treated by surgery or surgery in combination with radio- or chemotherapy. Excluding RMS, and independent of the primary metastatic status, single modality treatment did not prove to be less effective than multi-modality treatment on recurrence rate, DFI and OST.

A sub-analysis revealed that there was no significant difference in recurrence rate, DFI and OST after surgery alone compared with surgery and radiotherapy. The small number in the subgroups, however, could partly explain these findings.

While radiotherapy plays a major role in adjuvant treatment in extremity and trunk sarcomas, and adjuvant chemotherapy may add to local control,[6–10] in this retrospective study no proof for benefit of adjuvant treatment was found in primary treatment of pelvic sarcomas. This confirms findings in previous studies demonstrating surgery to be the only effective primary treatment in pelvic sarcomas and that adjuvant radiotherapy and chemotherapy did not seem to improve overall survival.[2,3] Although the role of adjuvant or neoadjuvant chemotherapy in the treatment of RMS in childhood is indisputable, in this study and in adulthood it is yet to be proven.

Pelvic surgery is limited because of the early involvement of adjacent pelvic structures, therefore surgery is often performed with minimal margins, which is reflected by the high incidence of local recurrence. The local recurrence rate seen in this study was 25% (6/24), and overall recurrence (local and distant) after primary treatment in patients with localized disease was seen in 15 of 24 (63%). Histology, grade (except for the low grade tumors), surgical margins and treatment modality did not seem to influence the frequency of recurrence. There was a high recurrence rate seen in patients with primary non-metastatic disease; multi-modality treatment approach, however, proved successful for these recurrences, considering the prolonged overall survival time.

The local recurrence rate in extremity and trunk sarcomas in non-metastatic disease is usually less than 20% and distant disease is often found first and mainly as pulmonary metastases.[5] The pathway of distant metastatic disease in pelvic sarcomas is also different from extremity sarcomas, since the majority are non-pulmonary metastases, as six of 11 patients developed distant metastases located in liver, skeletal bones or soft tissues.

In the MSKCC study, more low grade sarcomas were seen and grade and margins were considered prognostic factors. In that study, 49% died of disease, 5% were alive with evidence of disease and 36% were alive with no evidence of disease. Unlike this study, and unlike extremity and trunk sarcomas, in our study histology, grade and surgical margins did not show significant differences in recurrence rate, DFI and OST. However, we could not perform a multivariate analysis on prognostic factors considering the small number of patients in our study.

In our study the overall 5-year survival was 33%, which is comparable with previous studies where 20–60% was found.[1]

Our findings show resemblance to studies of retroperitoneal sarcomas, where the late presentation and frequent invasion of adjacent structures are also a main problem for achieving complete surgical resection. The most effective treatment modality for these tumors is complete surgical resection; chemotherapy has not proven to be effective and radiotherapy is limited by toxicity to adjacent structures. Patients with retroperitoneal sarcomas, however, often die of uncontrollable recurrent local disease,[11–13] whereas in this study metastatic disease was the main factor in fatal outcome. Surgical management of metastatic disease resulted in long-term survival in three patients in this study. In selected cases of pulmonary and liver metastases, surgical management of metastatic disease can result in 30–35% 5-year survival.[14–17]

### Conclusions

Pelvic sarcomas are difficult to diagnose because of their location, their late presentation and their rarity. Pelvic sarcomas, although a heterogeneous group of tumors, have specific characteristics, such as a high local recurrence rate and a high primary metastatic presentation rate. Primary metastatic disease proves to be an important poor prognostic factor, but adequate treatment of local disease and local recurrence can still result in a long-term successful outcome. Therefore it is of clinical importance that these sarcomas are considered as a possible diagnosis, despite their rarity in the pelvis!

Due to the heterogeneity of these tumors, the treatment approach may be various. In this study multi-modality treatment for most primary tumors did not show a significant benefit in recurrence rate, DFI and OST, when compared to single modality approach.

A prolonged DFI and OST was seen to be related to low grade only, but the small number of patients in this study may explain the lack of other prognostic factors.

The heterogeneity of the different types of sarcomas makes drawing clear conclusions difficult, but the differences with retroperitoneal, trunk and extremity sarcomas should be taken into consideration in the management of pelvic sarcomas.

### References

1. Herr HW. Sarcomas of the urinary tract. *Genitourinary Cancer Management*. Philadelphia, PA: Lea & Febiger, 1987; 259–70.
2. Russo P, Brady MS, Conlon K, Hadju SI, Fair WR, Herr HW, Brennan MF. Adult urological sarcoma. *J Urol* 1992; 47: 1032–7.
3. Zhang G, Chen KK, Manivel C, Fraley EE. Sarcomas of the retroperitoneum and genitourinary tract. *J Urol* 1989; 141: 1107–10.
4. de Saint Aubain Somerhausen N, Fletcher CDM. Soft tissue sarcomas: an update. *Eur J Surg Oncol* 1999; 25: 215–20.
5. Billingsley KG, Lewis JJ, Leung DH, Casper ES, Woodruff JM, Brennan MF. Multifactorial analysis of the survival of patients with distant metastases arising from primary extremity sarcoma. *Cancer* 1999; 85(2): 389–95.
6. Keus RB, Rutgers EJ, Ho GH, Gortzak E, Albus-Lutter CE, Hart AA. Limb-sparing therapy of extremity soft tissue sarcomas: treatment outcome and long-term functional results. *Eur J Cancer* 1994; 30A(10): 1459–63.
7. The Sarcoma Meta-analysis Collaboration. Adjuvant chemotherapy for localised resectable soft-tissue sarcoma of adult: meta-analysis of individual data. *Lancet* 1997; 350: 1647–54.
8. Gortzak E, Azzarelli A, Buesa J, Bramwell VH, van Coevorden F, van Geel AN, Ezzat A, Santoro A, Oosterhuis JW, van Glabbeke M, Kirkpatrick A, Verweij J. A randomised phase II study on neo-adjuvant chemotherapy for 'high-risk' adult soft-tissue sarcoma. *Eur J Cancer* 2001; 37(9): 1096–103.
9. Gortzak E, van Coevorden F. Soft tissue sarcoma: messages from completed randomized trials. *Eur J Surg Oncol* 1995; 21: 469–77.
10. Lewis JJ, Benedetti F. Adjuvant therapy for soft tissue sarcomas. *Surg Oncol Clin North Am* 1997; 6: 847–62.
11. Lewis JJ, Leung D, Woodruff JM, Brennan MF. Retroperitoneal soft-tissue Sarcoma. *Ann Surg* 1998; 228(3): 355–65.
12. van Doorn RC, Keus RB, Gallee MPWE, Hart AAM, Gortzak E, Rutgers EJT, van Coevorden F, Zoetmulder FAN. Resectable retroperitoneal soft tissue sarcomas: the effect of extent of resection and postoperative radiation therapy on local tumor growth. *Cancer* 1994; 73: 637–42.
13. van Dalen Th, Hoekstra HJ, van Geel AN, van Coevorden F, Albus-Lutter Ch, Slootweg PJ, Hennipman A, for the Dutch Soft Tissue Sarcoma Group. Locoregional recurrence of retroperitoneal soft tissue sarcoma: second chance of cure for selected patients. *Eur J Surg Oncol* 2001; 27: 564–8.
14. van Geel AN, van Coevorden F, Blankensteyn JD, Hoekstra HJ, Schuurman B, Bruggink EDM, Taat CW, Theunissen EBM. Surgical treatment of pulmonary metastases from soft tissue sarcomas: a retrospective study in the Netherlands. *J Surg Oncol* 1994; 56: 172–7.
15. van Geel AN, Hoekstra HJ, van Coevorden F, Meyer S, Bruggink EDM, Blankensteyn JD. Repeated metastasectomy for recurrent pulmonary metastatic soft tissue sarcoma. *Eur J Surg Oncol* 1994; 20: 436–41.
16. Van Geel AN, Pastorino U, Jauch KW, Judson IR, van Coevorden F, Buesa JM, Nielsen OS, Boudinet A, Tursz T, Schmitz PI. Surgical treatment of lung metastases: The European Organization for Research and Treatment of Cancer-Soft Tissue and Bone Sarcoma Group study of 255 patients. *Cancer* 1996; 77(4): 675–82.
17. van Ruth S, Mutsaerts E, Zoetmulder FA, van Coevorden F. Metastasectomy for liver metastases of non-colorectal primaries. *Eur J Surg Oncol* 2001; 27(7): 662–7.
18. Demetri GD, *et al*. Efficacy and safety of Imatinib mesylate in advanced gastrointestinal stromal tumors. *New Engl J Med* 2002; 347: 472–80.
